# Effect of SGLT inhibitors on weight and lipid metabolism at 24 weeks of treatment in patients with diabetes mellitus: A systematic review and network meta-analysis

**DOI:** 10.1097/MD.0000000000024593

**Published:** 2021-02-12

**Authors:** Mao-bing Chen, Hua Wang, Wei-yan Cui, Hua-lan Xu, Qi-han Zheng

**Affiliations:** a Department of Emergency; b Department of ICU, Wujin People's Hospital Affiliated with Jiangsu University and Wujin Clinical College of Xuzhou Medical University, Changzhou, Jiangsu, P.R. China.

**Keywords:** lipid metabolism, network meta-analysis, sodium-glucose transporter 1, sodium-glucose transporter 2, type 2 diabetes mellitus

## Abstract

**Background:**

The goals of improving quality of life and increasing longevity are receiving growing amounts of attention. Body weight and lipid metabolism are closely related to various complications of diabetes. The aim of this study was to rank SGLT inhibitors according to their efficacy with regard to weight and evaluate the effect of SGLT inhibitors on lipid metabolism at 24 weeks of treatment.

**Methods:**

The Web of Science, PubMed, Cochrane Library, Embase, and Clinical Trials databases were electronically searched to collect randomized controlled trials involving patients with type 2 diabetes mellitus through June 2020. Two researchers independently screened and evaluated the selected studies and extracted the outcome indexes. ADDIS 1.16.5 and STATA 16 software were used to perform the network meta-analysis and draw the plots.

**Results:**

Ultimately, 36 studies were selected and included in this study. We found that all SGLT inhibitors were effective at reducing weight; canagliflozin was the most effective. SGLT inhibitors and placebo were not associated with significantly different serum cholesterol levels. SGLT inhibitors lowered serum triglyceride levels and increased serum high-density and low-density lipoprotein cholesterol levels. SGLT inhibitors also reduced the level of alanine aminotransferase.

**Conclusions:**

SGLT inhibitors can bring about weight loss in patients with T2DM and can also improve lipid metabolism. Therefore, patients with hyperlipidemia who have been unsuccessful at losing weight should consider taking SGLT inhibitors. In addition, SGLT inhibitors are hepatoprotective and appear to be safe for patients with mild to moderate liver dysfunction.

**Trial Registration:**

CRD42020198516.

## 1 Introduction

Diabetes mellitus (DM), commonly referred to as diabetes, is a group of metabolic disorders characterized by long-term high blood sugar levels.^[1]^ Most diabetes patients have type 2 diabetes mellitus (T2DM). As of 2019, an estimated 463 million people had diabetes worldwide.^[2]^ The incidence of diabetes grows annually.^[3,4]^ In 2019, diabetes caused approximately 4.2 million deaths, and it was the seventh leading cause of death.^[5,6]^ Therefore, drugs for treating diabetes have long been the focus of attention of various research teams.

Sodium-dependent glucose transporter (SGLT) inhibitors are a new class of drugs for the treatment of T2DM.^[7]^ There are five main SGLT inhibitors, namely, dapagliflozin (DAPA), canagliflozin (CANA), empagliflozin (EMPA), ertugliflozin (ERTU), and sotagliflozin (SOTA).^[8]^ Among them, only SOTA is a dual SGLT-1/2 inhibitor. These drugs reduce blood glucose levels by interfering with SGLT, reducing glucose absorption or increasing glucose excretion. In previous studies, we verified the efficacy of SGLT inhibitors for the treatment of T2DM.^[9]^ However, with regard to their effects on lipid metabolism in patients with T2DM, a reliable systematic review has not yet been performed.

Obesity, especially central obesity, affects blood lipid metabolism and exacerbates insulin resistance in diabetic patients,^[10]^ and abnormal lipid metabolism is an important risk factor for complications of diabetes.^[11]^ In central obesity, fat usually accumulates in the liver. Liver fat deposits and high blood sugar have toxic effects on liver cells, and the main site of lipid metabolism and drug metabolism is the liver; therefore, it is necessary to evaluate the hepatic function of patients taking oral SGLT inhibitors. Studying the effect of SGLT inhibitors on lipid metabolism could elucidate their relationship with diabetes complications. The focus of this study was to evaluate the effect of SGLT inhibitors on lipid metabolism in patients with T2DM.

## 2 Methods

The original plan of this study was to perform a network meta-analysis. After data extraction, it was found that the data for the secondary outcomes were insufficient. Therefore, a common meta-analysis was performed for the levels of triglycerides, cholesterol, HDL-C, LDL-C, and ALT.

### 2.1 Design and registration

A network meta-analysis was conducted to evaluate the efficacy and safety of SGLT inhibitors in patients with T2DM. This protocol was registered with the International Prospective Register of Systematic Reviews (PROSPERO), with registration number: CRD42020198516 (https://www.crd.york.ac.uk/PROSPERO). No ethics approval was required because this study used data that were already in the public domain.

### 2.2 Study selection

#### 2.2.1 Study type

This network meta-analysis quantitatively analyzed data from randomized controlled trials (RCTs).

#### 2.2.2 Study subjects

The subjects of this study were patients with T2DM. There were no restrictions on age, weight, HbA1c level, drug history, etc. However, patients with serious underlying acute or chronic diseases and heart or kidney failure were excluded.

#### 2.2.3 Intervention measures

This network meta-analysis only included single-drug studies, and studies involving drug combinations were not included. Five drugs were included in this study, and each drug can be administered in two different doses; therefore, there were 10 interventions. In addition to the placebo group, a total of 11 interventions were included.

#### 2.2.4 Outcome Indicators

The primary outcome indicator was weight. Other outcome indicators included changes in levels of ALT, cholesterol, triglycerides, high-density lipoprotein/high-density lipoprotein cholesterol (HDL/HDL-C) and low-density lipoprotein/low-density lipoprotein cholesterol (LDL/LDL-C).

In a preliminary analysis, it was found that weight was strongly affected by the duration of treatment. To reduce the heterogeneity among the studies, limitations were placed on the duration of treatment. Results after treatment for longer than 24 weeks were included because we found that after 24 weeks, weight remained relatively stable. Therefore, we chose 24 weeks (±2 weeks) as the timepoint for data selection.

#### 2.2.5 Exclusion criteria

Studies with data that could not be extracted or utilized, studies with animal experiments, and literature reviews were excluded.

### 2.3 Data sources and searches

**Figure 1:**
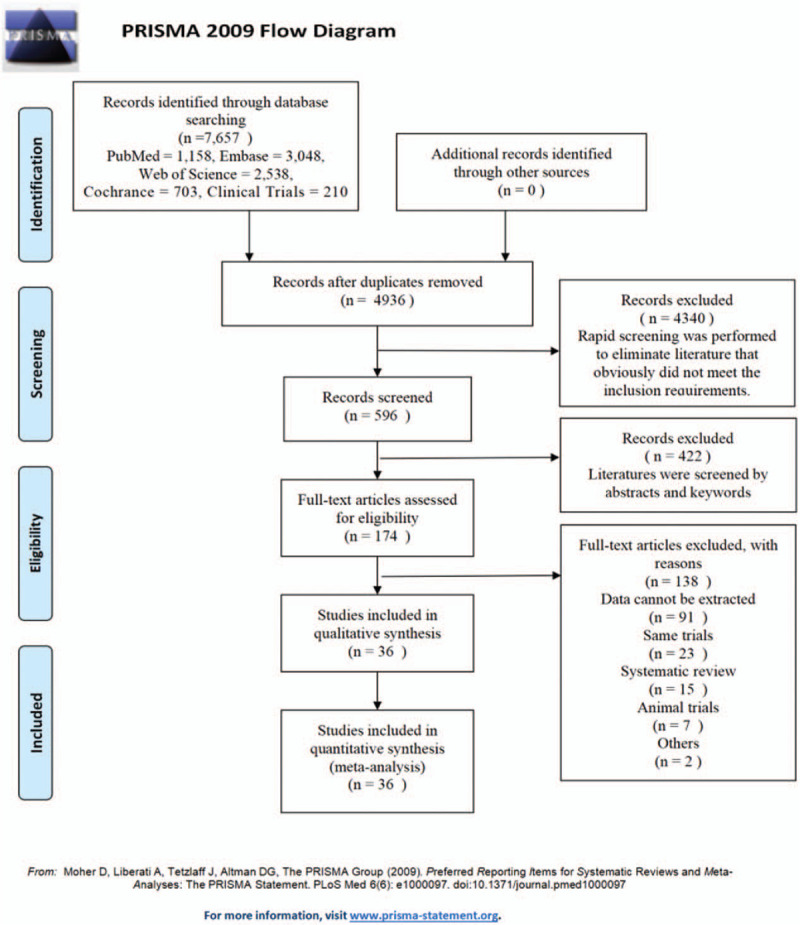
PubMed database retrieval strategy and PRISMA flow diagram.

We searched for publications through June 2020 in the following databases: Web of Science, PubMed, the Cochrane Library, EMBASE, and Clinical Trials. The search terms included “SGLT,” “diabetes,” and “mellitus.” In Figure 1, we use the PubMed database as an example.

### 2.4 Study screening, data extraction, and assessment of the risk of bias

Data were collected independently by two researchers. The unqualified studies were eliminated, and the qualified studies were selected after reading the title, abstract and full text. Then, the research data were extracted and checked, disagreements were discussed, and a decision was made regarding study inclusion by the authors. The extracted data included the following:

1. the basic information about the study, including title, author and year of publication;
2. the characteristics of the included study, consisting of the study duration, the sample sizes of the test group and the control group, and the intervention measures
3. the outcome indicators and data; and
4. the information needed to assess the risk of bias.

The risk of bias in the included studies was assessed using the RCT bias risk assessment tool recommended in the Cochrane Handbook for Systematic Reviews of Interventions (5.1.0).^[12]^

### 2.5 Statistical analysis

This network meta-analysis was performed using the Bayesian method. STAT MP 16 and ADDIS 1.16.5 software were used to draw the plots and perform the network meta-analysis, and RevMan 5.4 software was used for the common meta-analysis. The continuous variables are expressed as the mean difference (MD) as an effect indicator. Effect estimates and 95% confidence intervals (CIs) were calculated. A network meta-analysis was performed for weight, and a common meta-analysis was performed for other outcome indexes due to the lack of data. A random effects model was used in the network meta-analysis, and a fixed model was used in the common meta-analysis. The core results of the network meta-analysis included a network evidence plot, network SUCRA plot, pairwise comparison plot and network node-splitting analysis of inconsistency. If an inconsistency was observed, the cause was identified, explained and analyzed, and the inconsistency model was used for analysis. Finally, a funnel plot was drawn if there were more than 15 studies. The significance level was set at α = 0.05.

## 3 Results

### 3.1 Included studies and patients

From the databases, we retrieved a total of 7,657 studies. Ultimately, 36 studies^[13–48]^ were selected and included. No gray literature was included in this study. The specific flow diagram is shown in Figure 1. When the data from the included studies were pooled, the total number of enrolled patients was 17,561. In each study, the characteristics of the patients in the groups were similar.

We originally planned to include five well-known SGLT inhibitors in this study. However, the data in the SOTA-related RCTs did not meet the inclusion criteria. Therefore, there are no SOTA data included in these results.

### 3.2 Characteristics of the included studies and quality assessment

All included studies were RCTs. The basic characteristics and quality assessment of the studies are presented in Table 1.

**Table 1:** The basic characteristics and quality assessment of the studies.

						Intervening Measure			Sample size			Literature quality score						
Author	Year	No. of trial	The country or region of the first author	Background	Time point of data extraction	Group-1	Group-2	Group-3	Group-1	Group-2	Group-3	Random sequence generation	Allocation concealment	Blinding of participants and personnel	Blinding of outcome assessment	Incomplete outcome data	Selective reporting	Other bias
Aronson, R.	2018	NCT01958671	North America	Diet and Exercise	26 weeks	ERTU 5mg	ERTU 15mg		156	152		Low risk	Low risk	Low risk	Low risk	Unclear	Low risk	Low risk
Bailey, C. J.	2010	NCT00528879	UK	MET	24 weeks	DAPA 5mg	DAPA 10mg	PLA	137	135	137	Low risk	Low risk	Low risk	Low risk	Low risk	Low risk	Low risk
Bailey, C. J.	2012	–	UK	Diet and Exercise	24 weeks	DAPA 5mg	PLA		68	68		Low risk	Low risk	Low risk	Low risk	Low risk	Low risk	Unclear
Bode, Bruce	2013	NCT01106651	US	No limit	26 weeks	CANA 100mg	CANA 300mg	PLA	241	236	237	Low risk	Low risk	Low risk	Low risk	Low risk	Low risk	Unclear
Bolinder, J.	2014	NCT00855166	Sweden	MET	26weeks	DAPA 10mg	PLA		89	91		Low risk	Low risk	Low risk	Low risk	Low risk	Low risk	Unclear
Dagogo-Jack, S.	2018	NCT02036515	US	MET and SITA	26 weeks	ERTU 5mg	ERTU 15mg	PLA	156	153	153	Low risk	Low risk	Low risk	Low risk	Low risk	Low risk	Unclear
Ferrannini, E.	2010	NCT00528372	Italy	Diet and Exercise	24 weeks	DAPA 5mg	DAPA 10mg	PLA	64	70	75	Low risk	Low risk	Low risk	Low risk	Unclear	Low risk	Unclear
Forst, T.	2014	NCT01106690	Germany	MET and pioglitazone	26 weeks	CANA 100mg	CANA 300mg	PLA	113	114	115	Low risk	Low risk	Low risk	Low risk	Low risk	Low risk	Unclear
Hadjadj, S.	2016	NCT01719003	France	No limit	24 weeks	EMPA 10mg	EMPA 25mg		169	164		Low risk	Low risk	Low risk	Low risk	Low risk	Low risk	Unclear
Haering, H. U.	2015	NCT01289990	Germany	MET and SU	26 weeks	EMPA 10mg	EMPA 25mg	PLA	225	216	225	Low risk	Low risk	Low risk	Low risk	Unclear	Unclear	Unclear
Haering, H. U.	2014	NCT01159600	Germany	Diet and Exercise	24 weeks	EMPA 10mg	EMPA 25mg	PLA	217	213	207	Low risk	Low risk	Low risk	Low risk	Low risk	Low risk	Unclear
Hollander, P.	2018	NCT01999218	US	MET	26 weeks	ERTU 5mg	ERTU 15mg		448	440		Low risk	Low risk	Low risk	Low risk	Unclear	Low risk	Unclear
Jabbour, Serge A.	2018	NCT00984867	US	SITA and/or MET	24 weeks	DAPA 10mg	PLA		223	224		Low risk	Low risk	Low risk	Low risk	Unclear	Low risk	Unclear
Ji, L.	2019	NCT02630706	China	MET	26 weeks	ERTU 5mg	ERTU 15mg	PLA	170	169	167	Low risk	Low risk	Low risk	Low risk	Low risk	Low risk	Unclear
Kadowaki, T.	2015	NCT01193218	Japan	Diet and Exercise	26 weeks	EMPA 10mg	EMPA 25mg		267	265		Low risk	Low risk	Low risk	Low risk	Low risk	Low risk	Unclear
Kadowaki, T.	2017	NCT02354235	Japan	Teneligliptin	24 weeks	CANA 100mg	PLA		70	68		Low risk	Low risk	Low risk	Low risk	Low risk	Low risk	Unclear
Kovacs, C. S.	2015	NCT01210001	Canada	MET	24 weeks	EMPA 10mg	EMPA 25mg	PLA	165	168	165	Low risk	Low risk	Low risk	Low risk	Unclear	Low risk	Unclear
Lavalle-Gonzalez, F. J.	2013	NCT01106677	Mexico	MET	26 weeks	CANA 100mg	CANA 300mg		368	367		Low risk	Low risk	Low risk	Low risk	Low risk	Low risk	Unclear
Lewin, A.	2015	NCT01422876	US	Diet and Exercise	26 weeks	EMPA 10mg	EMPA 25mg		132	133		Low risk	Low risk	Low risk	Low risk	Unclear	Low risk	Unclear
Mathieu, C.	2015	NCT01646320	Romania	MET and Saxagliptin	24 weeks	DAPA 10mg	PLA		160	160		Low risk	Low risk	Low risk	Low risk	Unclear	Low risk	Unclear
Matthaei, S.	2015	NCT01392677	Germany	MET and SUL	24 weeks	DAPA 10mg	PLA		108	108		Low risk	Low risk	Low risk	Low risk	Unclear	Low risk	Unclear
Merker, L.	2015	NCT01619059	Denmark	MET	26 weeks	EMPA 10mg	EMPA 25mg	PLA	217	213	207	Low risk	Low risk	Low risk	Low risk	Low risk	Low risk	Unclear
Pratley, R. E.	2018	NCT02099110	US	MET	26 weeks	ERTU 5mg	ERTU 15mg		250	248		Low risk	Low risk	Low risk	Low risk	Unclear	Low risk	Unclear
Roden, M.	2013	NCT01177813	Germany	Diet and Exercise	24 weeks	EMPA 10mg	EMPA 25mg	PLA	224	224	228	Low risk	Low risk	Low risk	Low risk	Low risk	Low risk	Unclear
Romera, I.	2016	–	Spain	MET or SUL and so on.	24 weeks	EMPA 10mg	EMPA 25mg	PLA	607	597	597	Low risk	Low risk	Low risk	Low risk	Unclear	Low risk	Unclear
Rosenstock, J.	2016	NCT01809327	US	Diet and Exercise	26 weeks	CANA 100mg	CANA 300mg		237	238		Low risk	Low risk	Low risk	Low risk	Low risk	Low risk	Unclear
Rosenstock, J.	2018	NCT02033889	US	MET	26 weeks	ERTU 5mg	ERTU 15mg	PLA	207	205	209	Low risk	Low risk	Low risk	Low risk	Low risk	Low risk	Unclear
Softeland, E.	2017	NCT01734785	Norway	Linagliptin and MET	24 weeks	EMPA 10mg	EMPA 25mg	PLA	109	110	108	Low risk	Low risk	Low risk	Low risk	Low risk	Low risk	Unclear
Sone, H.	2020	NCT02589639	Japan	insulin with or without one drug	26 weeks	EMPA 10mg	EMPA 25mg	PLA	86	90	90	Low risk	Low risk	Low risk	Low risk	Unclear	Low risk	Unclear
Stenlof, K.	2013	NCT00680745	Poland	Diet and Exercise	26 weeks	CANA 100mg	CANA 300mg	PLA	195	197	192	Low risk	Low risk	Low risk	Low risk	Low risk	Low risk	Unclear
Strojek, K.	2011	NCT00680745	Poland	Glimepiride	24 weeks	DAPA 5mg	DAPA 10mg	PLA	142	151	145	Low risk	Low risk	Low risk	Low risk	Low risk	Low risk	Unclear
Terra, S. G.	2017	NCT01958671.	US	Diet and Exercise	26 weeks	ERTU 5mg	ERTU 15mg	PLA	156	152	153	Low risk	Low risk	Low risk	Low risk	Unclear	Low risk	Unclear
Wilding, J. P.	2013	NCT01106625	UK	MET and SUL	26 weeks	CANA 100mg	CANA 300mg	PLA	157	156	156	Low risk	Low risk	Low risk	Low risk	Low risk	Low risk	Unclear
Wilding, J. P.	2012	NCT00673231	US	High doses of insulin	24 weeks	DAPA 5mg	DAPA 10mg	PLA	211	194	193	Low risk	Low risk	Low risk	Low risk	Low risk	Low risk	Unclear
Yang, W.	2016	NCT01095666	China	MET	24 weeks	DAPA 5mg	DAPA 10mg	PLA	147	152	145	low risk	low risk	low risk	low risk	low risk	unclear	unclear
Yang, W.	2018	NCT02096705	China	Insulin with or without oral antihyperglycemic drugs	24 weeks	DAPA 10mg	PLA		139	133		Low risk	Low risk	Low risk	Low risk	Low risk	Unclear	Unclear

### 3.3 Network meta-analysis results

#### 3.3.1 Weight

**Figure 2:**
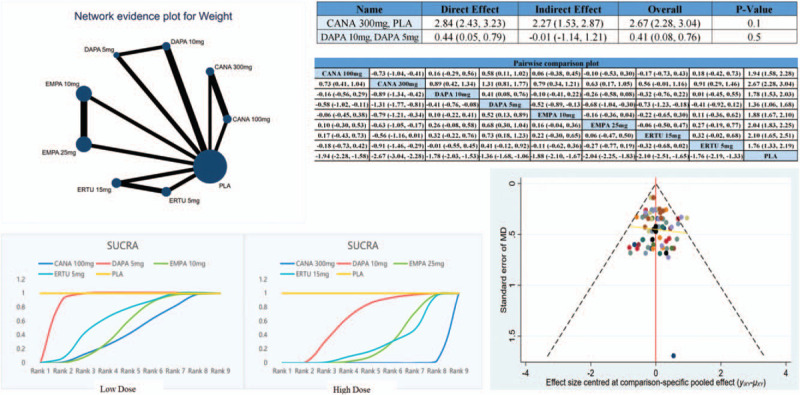
The core network analysis results for weight.

Thirty-six studies reported comparisons of weight. The core results of the network analysis are shown in Figure 2. According to the node-splitting analysis, the consistency model adopted in this study was reliable. The pairwise comparison plot shows the results of the comparisons between all the included interventions. Among the four SGLT-2 inhibitors, the SUCRA plot shows that 300 mg CANA (high dose) and 100 mg CANA (low dose) should be the most effective. Funnel plots were generated. The funnel plots were bilaterally symmetrical, and most studies fell within the 95% confidence interval. These results suggest that this study has no clear publication bias.

### 3.4 Common meta-analysis results

#### 3.4.1 Cholesterol

**Figure 3:**
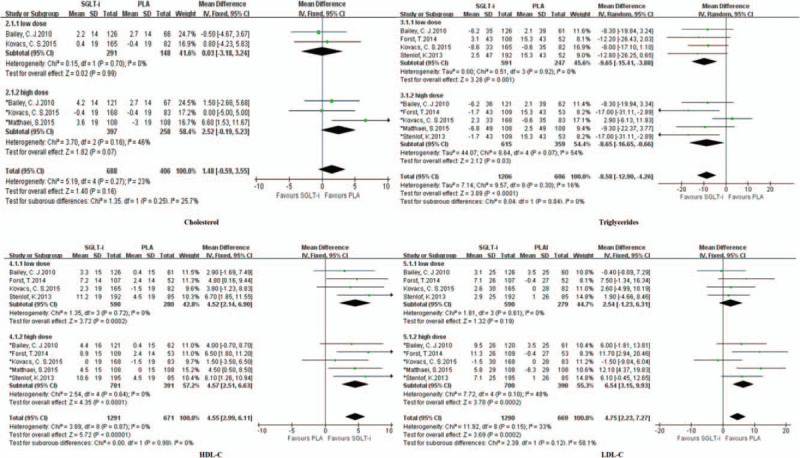
Forest plot comparing lipid metabolism between the SGLT inhibitor and placebo groups.

Three studies reported differences in cholesterol between the SGLT inhibitor group and the placebo group. A fixed-effect model was adopted; as the dose of SGLT inhibitors increased, serum cholesterol also increased (low dose: *I*^2^ = 0% [MD = 0.03, 95% CI (−3.18, 3.24), *P* = .99]; high dose: *I*^2^ = 46% [MD = 2.52, 95% CI (−0.19, 5.23), *P* = .07]) (Fig. 3, upper left).

#### 3.4.2 Triglyceride

Five studies reported differences in triglyceride levels between the SGLT inhibitor group and the placebo group. A random effect model was adopted; as the dose of SGLT inhibitors increased, the serum triglyceride level decreased (low dose: *I*^2^ = 0% [MD = −9.65, 95% CI (−15.41, −3.88), *P* = 0.001]; high dose: *I*^2^ = 54% [MD = −8.65, 95% CI (−16.65, −0.66), *P* = .03]) (Fig. 3, upper right).

#### 3.4.3 HDL/HDL-C

Five studies reported differences in HDL/HDL-C levels between the SGLT inhibitor group and the placebo group. A fixed effect model was adopted; compared with the placebo, oral SGLT inhibitors were associated with increased serum HDL/HDL-C levels (low dose: *I*^2^ = 0% [MD = 4.52, 95% CI (2.14,6.90), *P* = .0002]; high dose: *I*^2^ = 0% [MD = 4.57, 95% CI (2.51, 6.63), *P* < 0.0001]) (Fig. 3, lower left).

#### 3.4.4 LDL/LDL-C

Five studies reported differences in LDL/LDL-C levels between the SGLT inhibitor group and the placebo group. A fixed effect model was adopted; as the dose of SGLT inhibitors increased, the serum LDL/LDL-C level also increased (low dose: *I*^2^ = 0% [MD = 2.54, 95% CI (−1.23,6.31), *P* *=* 0.19]; high dose: *I*^2^ = 48% [MD = 6.54, 95% CI (3.15, 9.93), *P* = .0002]) (Fig. 3, lower right).

#### 3.4.5 ALT

**Figure 4:**
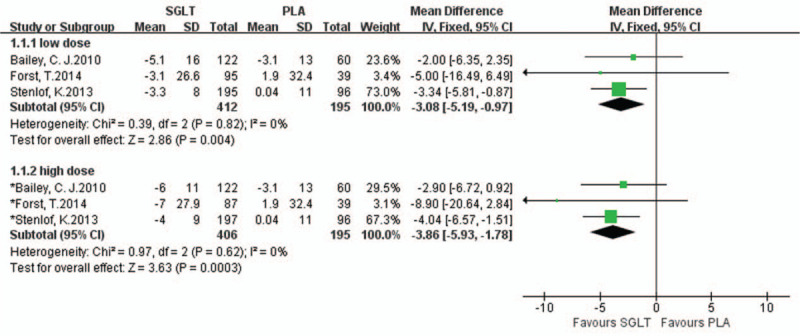
Forest plot comparing the ALT levels between the SGLT inhibitor and placebo groups.

Three studies reported differences in ALT levels between the SGLT inhibitor group and the placebo group. A fixed effect model was adopted; compared with the placebo, oral SGLT inhibitors were associated with decreased serum ALT levels (low dose: I^2^ = 0% [MD = −3.08, 95% CI (−5.19, −0.97), *P* = .004]; high dose: *I*^2^ = 0% [MD = −3.86, 95% CI (−5.93, −1.78), *P* = .0003]) (Fig. 4).

## 4 Discussion

Since no studies on dual SGLT-1/2 inhibitors were included in this study, these results only pertain to SGLT-2 inhibitors. RCTs on SOTA were excluded because the duration of the intervention did not meet the inclusion criteria.^[49]^

Based on this network meta-analysis, we believe that SGLT-2 inhibitors effectively induce weight loss in patients with T2DM; CANA is the most effective, and DAPA is the least effective. Second, SGLT-2 inhibitors can reduce triglyceride levels and increase both HDL-C and LDL-C levels. Finally, SGLT2 inhibitors can decrease serum ALT levels and may have a protective effect on the liver.

**Figure 5:**
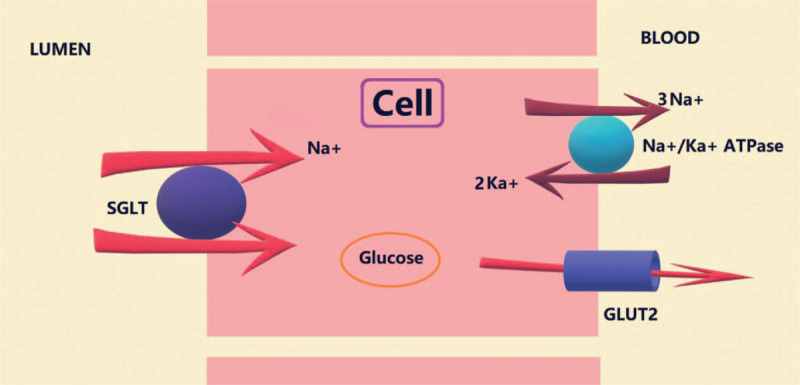
Mechanism of action of SGLT proteins in cells.

The main function of SGLT is to reabsorb glucose. The sodium–potassium pump consumes ATP and transfers Na^+^ to the outside of the cell, causing a decrease in the intracellular Na^+^ concentration. The Na^+^ in glomerular filtrate (or in intestinal juice) enters the cell along the concentration gradient, and glucose is brought into the cell concurrently by the action of sodium-dependent glucose transporters. This is the mechanism by which SGLT reabsorbs glucose^[50]^ (Fig. 5).

Once the mechanism of SGLT is understood, the mechanism by which SGLT inhibitors control blood sugar in patients with T2DM is clear. In the real world, many people with T2DM also have hyperlipidemia; accordingly, T2DM and hyperlipidemia are usually considered sister diseases, and hyperlipidemia is believed to be a secondary disease of T2DM.^[51]^ According to a cross-sectional study, approximately 60% of diabetic patients have hypertriglyceridemia.^[52]^ The state of hyperlipidemia substantially increases patients’ cardiovascular risk.^[53,54]^ Compared with other hypoglycemic drugs, the advantage of SGLT-2 inhibitors is that they induce weight loss in patients with T2DM and simultaneously have a beneficial effect on lipid metabolism.^[55]^ The results of our study corroborate this conclusion.

SGLT-2 inhibitors probably induce weight loss by reducing the body's total energy intake and promoting osmotic diuresis. However, the effects of SGLT inhibitors on lipid metabolism might be carried out in the following ways. Diabetic dyslipidemia is characterized by elevated serum triglyceride levels, decreased serum high-density lipoprotein cholesterol (HDL-C) levels, and predominant atherosclerotic low-density lipoprotein (LDL) particles.^[56]^ There is a dual effect of SGLT-2 inhibitors on lipids: on the one hand, SGLT-2 inhibitors might increase the breakdown of fats, leading to increases in liver levels of cholesterol substrate and hepatic hydroxy-methyl-glutaryl CoA (HMG-CoA). This, in turn, would increase cholesterol synthesis, decrease the activity of LDL receptors and finally lead to an increase in serum LDL-C level. On the other hand, SGLT-2 inhibitors could reduce the systemic toxicity of glucose, thereby reducing triglyceride synthesis in the liver and increasing the breakdown of triglycerides. This, in turn, would reduce the serum triglyceride level and ultimately lead to an increased serum HDL-C level.^[57]^

SGLT inhibitors increase the levels of glucagon-like peptide 1 (GLP-1), one of the brain-gut peptides. GLP-1 can promote the glucose-dependent production and release of insulin and inhibit glucagon secretion, gastric emptying, food intake, and nutrient absorption.^[58]^ Thus, GLP-1 can reduce blood sugar and control weight, similar to SGLT inhibitors. In a randomized clinical trial by Zambrowicz B, 300 mg of LX4211 (SOTA) was given to T2DM patients, and the level of GLP-1 substantially increased.^[59]^

This study verifies by serology that SGLT inhibitors can improve the lipid metabolism of patients with T2DM. In an epidemiological investigation, SGLT inhibitors improved atherosclerosis and reduce the risk of cardiovascular and cerebrovascular events.^[60]^ The Comparative Effectiveness of Cardiovascular Outcomes in New Users of SGLT-2 Inhibitors (CVD-REAL) study results were presented at the 66th Annual Meeting of the American College of Cardiology. The study included 300,000 patients with T2DM. Compared with other hypoglycemia drugs, SGLT-2 inhibitors reduced all-cause mortality by 51% and heart failure in-hospital mortality by 39%. The mechanism underlying the protective effect of SGLT inhibitors is currently unclear. In addition to improving lipid metabolism, they may also have beneficial effects on myocardial fibers by activating the Stat3 signaling pathway^[61]^ or by inhibiting the exchange of Na+/H+ in cardiomyocytes, reducing the concentration of cytoplasmic Na+ and Ca2+, and increasing the concentration of mitochondrial Ca2+, thereby exerting a protective effect on the myocardium.^[62]^ It is not clear whether the SGLT-2 protein is expressed in the heart.

The limitations of this network meta-analysis are as follows:

1. The laboratory examination data related to lipid metabolism were limited, making it impossible to conduct a network meta-analysis for all the outcome. A common meta-analysis was performed instead.
2. The results of laboratory tests for triglycerides and cholesterol are highly dynamic, which could have interfered with the results.
3. Different types of SGLT inhibitors might have different effects on the levels of triglyceride, cholesterol and ALT.

## 5 Conclusions

SGLT inhibitors can induce weight loss in patients with T2DM and improve lipid metabolism. Therefore, diabetic patients with uncontrolled weight should consider taking SGLT inhibitors. In addition, they are safe in patients with mild to moderate liver dysfunction.

## Author contributions

**Conceptualization:** Mao-bing Chen, Wei-yan Cui, Qi-han Zheng.

**Data curation:** Mao-bing Chen, Qi-han Zheng.

**Methodology:** Wei-yan Cui, Qi-han Zheng.

**Software:** Mao-bing Chen, Hua Wang, Hua-lan Xu.

**Supervision:** Mao-bing Chen, Hua-lan Xu.

**Writing – original draft:** Mao-bing Chen, Hua Wang, Wei-yan Cui, Hua-lan Xu, Qi-han Zheng.

**Writing – review & editing:** Mao-bing Chen, Hua Wang.

## Abbreviations

Abbreviations: CANA = canagliflozin, CI = confidence interval, DAPA = dapagliflozin, DM = diabetes mellitus, EMPA = empagliflozin, ERTU = ertugliflozin, HDL/HDL-C = high-density lipoprotein/high-density lipoprotein cholesterol, HMG-CoA = hydroxy-methyl-glutaryl CoA, LDL/LDL-C = low-density lipoprotein/low-density lipoprotein cholesterol, MD = mean difference, PROSPERO = International Prospective Register of Systematic Reviews, RCTs = randomized controlled trials, SGLT = sodium-dependent glucose transporter, SOTA = sotagliflozin, T2DM = type 2 diabetes mellitus.
